# Identity and entitlement in accounts of (morally) normative and informational social influence for sustainability

**DOI:** 10.1111/bjso.70061

**Published:** 2026-03-08

**Authors:** Liz Cooper

**Affiliations:** ^1^ School of Philosophy, Psychology and Language Sciences University of Edinburgh Edinburgh UK; ^2^ School of Social Sciences Heriot‐Watt University Edinburgh UK

**Keywords:** discursive psychology, identity, moral entitlement, normative and informational social influence, product design, social influence, sustainability

## Abstract

Recent discursive psychology research has sought to respecify social influence as a discursive accomplishment and has also begun to identify how the psychological thesaurus is used to portray social influence in situated talk. The present paper contributes to this project by examining how social influence is depicted in accounts of influencing others in professional settings. Product designers' portrayals of influencing decision‐makers to prioritize environmental sustainability, collected through semi‐structured research interviews and from conference panel discussions, are analysed. Two recurring ways of constructing social influence are found—morally normative influence involving effort and force against resistance (‘pushing’) and informational influence through educating. The analysis shows how these depictions of influencing represent situated identity work. Two contributions are made to understanding ways of depicting social influence in professional settings. First, whether people claim entitlement to influence others at work is highlighted as a key element in how an influencer's identity is portrayed. Second, the participants' orientation to moral norms, not just social norms, is offered to extend to the concept of normative social influence in the context of sustainability. Implications for understanding how people relate to the shared moral challenge of environmental sustainability in different interactional contexts are discussed.

## INTRODUCTION

This paper analyses accounts of social influence towards environmentally sustainable business practices, offering new insights into the discursive practices used to characterize different ways of influencing others. In psychology, social influence has been classified into different types: conformity, obedience, compliance and persuasion (Cialdini, 2009), where people are assumed to have different starting positions on a particular course of action or issue. Experimental methods have explored the conditions under which different types of influence occur (Cialdini, 2009). Conformity with others' actions and group norms has been demonstrated through showing that participants often adopt incorrect answers when confederates do so (Asch, 1951; Duderstadt et al., 2024). Obedience, involving following orders given by authority figures, has been shown through Milgram's (1974) experiments involving directing participants to give electric shocks to learners. Compliance, involving following instructions without necessarily changing underlying attitudes, has been demonstrated in field experiments showing that people are more likely to grant a request if it follows a less onerous, related one (Dolinski, 2015; Freedman & Fraser, 1966). Persuasion, defined as involving a change to attitudes, beliefs or behaviours, has been demonstrated through the use of persuasive messaging that appeals to rational or emotional arguments, often examining persistence of attitude change over time (Compton, 2025; Hovland, 1951).

Beyond experimental settings, these different types of social influence are prevalent in everyday life, with both positive and negative effects. Environmental sustainability represents a context in which social influence is necessary for shifting how societies use natural resources to reduce pollution and waste. People, as private individuals and as professionals, can be asked to conform to shifting societal norms regarding reducing waste, required to comply with new environmental regulations, or persuaded to actively adopt more environmentally friendly practices. As a shared moral issue facing societies and organizations today (Ha‐Brookshire, 2017; Jordan & Kristjánsson, 2017; Marques, 2016), sustainability also invites moral arguments for persuasion, alongside rational and emotional appeals. Messaging strategies advocated for sustainable behaviour change include storytelling, humanizing climate change impacts, appealing to emotions and highlighting benefits for individuals rather than for society as a whole (Godemann, 2021; Hardeman et al., 2017; Villarino & Font, 2015).

To better understand the nuances of social influence in practice, discursive psychology (DP) advocates a closer focus on language use to observe both how influence is accomplished through talk‐in‐interaction (Gibson & Smart, 2017; Humă et al., 2020b) and how people describe and portray different strategies for influencing within situated interactions (Humă et al., 2020a). This paper uses a DP approach to study how product designers portray social influence for sustainability when constructing accounts of their work in research interviews and conference panel discussions. The analysis shows how matters of identity and entitlement in professional settings are negotiated within the situated interactions through depicting either morally normative or informational influencing for sustainability. The remainder of this paper reviews theories of normative and informational social influence, outlines the discursive re‐specification of social influence and then presents the study context and analysis.

## NORMATIVE AND INFORMATIONAL SOCIAL INFLUENCE IN SOCIAL PSYCHOLOGY

To explain the motives people may have for allowing themselves to be influenced, Deutsch and Gerard (1955) developed the theories of normative and informational social influence. Normative social influence, defined as ‘an influence to conform with the positive expectations of another’ (Deutsch & Gerard, 1955, p. 629), involves compliance with social norms to gain acceptance in a group or avoid disapproval from others. This is demonstrated in their recreations of Asch's (1951) experiments in which participants are seen to agree with confederates that lines are longer than they actually are. People's own judgements are said to be overridden, either through witnessing or hearing about others' acts (Nolan et al., 2008). In workplaces, normative social influence is said to be at play when employees conform with organizational norms (Schneider et al., 2013).

Field studies have shown that normative social influence can effectively promote environmental behaviour. Schultz (1999) found that telling people about how much their neighbours recycled increased their own recycling rates. Goldstein et al. (2008) showed that providing messaging about other hotel guests reusing their towels (rather than requesting daily washes) influenced people to reuse their own towels. Nolan et al. (2008) found a strong correlation between believing other people were conserving energy at home and people's own energy saving practices. This normative social influence was found to be much more effective than asking people to save energy on moral grounds (Nolan et al., 2008). The term *normative* in psychology relates to social norms. However, in philosophy and economics, *normative* refers to what should be, based on moral ideals and personal beliefs about desirable futures (Debru, 2011; Weston, 1994). This opens up the possibility of considering the morally normative dimension of social influence in sustainability contexts.

Informational social influence, which Deutsch and Gerard (1955, p. 629) define as ‘an influence to accept information obtained from another as evidence about reality’, is said to occur when people conform because they believe that others have relevant knowledge in an ambiguous situation, such as when a student's sociopolitical attitudes change based on their university studies (Guimond, 1997). People influencing for sustainability goals through providing information must therefore trust that the influencees view a situation as ambiguous and also believe that the influencers possess accurate information. Studies on informational social influence for sustainability show that providing information is effective when combined with integration into a social group (Estrada et al., 2017; Salazar et al., 2013), indicating that group norms and trust also matter.

## THE DISCURSIVE RE‐SPECIFICATION OF SOCIAL INFLUENCE

DP challenges experimental social psychology's treatment of social influence as one‐way messaging, arguing that this overlooks the complexities of real‐world interactional and interpersonal dynamics (Gibson & Smart, 2017; Humă et al., 2020b). In DP research, talk is treated as socially constructed within its interactional context and constructive of different conceptualisations of realities (Wiggins, 2017). Detailed analysis of the action‐orientation of talk and the discursive strategies used to achieve actions allows for the re‐specification of traditional cognitive psychology theories. For example, examining how people construct attitudes in a specific conversation may reveal contradictions and inconsistencies to achieve immediate goals such as positive identity portrayal or deflecting accountability (Potter & Wetherell, 1987). Rather than assuming that what people say represents stable, inner mental states, attitudes are respecified as discursivity constructed in context.

DP researchers have questioned the theoretical assumptions about how social influence operates, highlighting greater complexity when the phenomena are observed in real‐world settings. Gibson (2019b), reviewing recordings of Milgram's (1974) obedience experiments, has shown how obedience in practice is not necessarily about responding to the explicit orders of an authority figure, but is a product of implicit expectations of the wider social and interactional context in which it occurs. Hepburn and Potter (2011) have shown how parents use conditional threats at the dinner table to encourage children to obediently finish their meals and how children can circumvent threats rather than responding with explicit compliance or defiance. Humă et al. (2020b) have shown how persuasion between a sales agent and target takes place gradually and cannot be pinned down to specific utterances. Here, persuasion is not treated as involving attitude change since DP avoids making assumptions about inner mental states.

DP also has an over‐arching goal of studying the everyday use of the ‘psychological thesaurus’ ‐ how psychological language is used in everyday interactions for particular ends (Edwards & Potter, 2005, p. 241). The terms psychologists use to define types of social influence do not necessarily reflect the ways people talk about influencing in situated interactions. Humă et al. (2020a, p. 319) have recently studied ways in which social influence is characterized in talk, identifying what they describe as ‘vocabularies of social influence’. They analysed recordings of interactions including phone calls to mediation services, police interrogations and business‐to‐business cold calls, to identify instances where people talked about, or alluded to, persuasion (Humă et al., 2020a). Note the focus here was not on the attempts at persuasion themselves, but on how they were described to others. They found that different terms were used depending on the extent to which the speakers oriented to an entitlement to influence others' actions. For example, when talking about business‐to‐business sales tactics, ‘hoodwinking’ and ‘schmoozing’ were found to be used to avoid explicitly naming the goal as persuasion, which helps manage the moral dilemma of seeking to change someone else's behaviour thereby curtailing their autonomy (Humă et al., 2020a). Building on this work, the present paper identifies additional vocabularies used to characterize types of social influence, in accounts of influencing towards environmental sustainability in business settings.

## THE STUDY—ACCOUNTS OF SUSTAINABILITY‐RELATED DECISIONS AND INFLUENCE IN PRODUCT DESIGN

In the conversations analysed, designers give accounts of influencing towards more sustainable product design. Sustainability is commonly defined as comprising environmental, social and economic pillars (Purvis et al., 2019), although the environmental aspect is often foregrounded (Caradonna, 2022; Ruggerio, 2021). Product design and manufacturing make significant contributions to pollution and waste, and many efforts have been made to shift to more sustainable production (Keitsch, 2012; Schöggl et al., 2017). Models and tools have been developed to promote more sustainable product design, considering factors such as carbon emissions, recyclability, durability and costs (Hallstedt, 2017; Inoue et al., 2012; Kapelan et al., 2005; Wang et al., 2017).

Designers are often assumed to be key actors in the transition to more sustainable production and consumption (Chick & Micklethwaite, 2011; Fairs, 2019). However, they may lack final decision‐making power regarding how sustainable products are (Cooper, 2023; Mejía & Fischer, 2022). Rather than acting as individual decision‐makers, designers typically collaborate and negotiate with multiple stakeholders (Bucciarelli, 1988; Feng & Feenberg, 2008; Lloyd & Busby, 2003; Luck, 2015; van de Poel et al., 2015). Designers might therefore seek to influence design decisions towards sustainable solutions (Fry, 2004; van de Poel & Verbeek, 2006). Yet clients or managers may resist such efforts on economic grounds, since the cheapest materials are not likely to be the more environmentally sustainable ones (Azevedo et al., 2022). While the notion of a sustainability champion or insider change agent working to influence from within is widely studied in business literature (Andersson & Bateman, 2000; Heucher et al., 2024), there is little prior empirical research aimed at understanding how designers articulate their roles as involving influencing for sustainability.

### Data collection methods

Given the expectations about designers' roles in advancing sustainability, this study examines instances in which product designers are asked questions about sustainability‐related decision‐making, both in interviews and in conference panel discussions. The use of co‐constructed interview data in DP has been the subject of considerable debate, since there is a likelihood of ‘flooding of the interview with social science agendas’ by the interviewer, and both interviewer and interviewee are likely to be managing their stake and interest in the topic discussed (Potter & Hepburn, 2005, p. 299). Nonetheless, many DP studies have produced insightful findings from analysing interview data, taking into account the nature of research interviews, contributing to topics such as identity construction, politeness and how accountability is navigated when facing direct questions (Rapley, 2015; Tracy & Robles, 2010; Widdicombe, 2017). DP analysis of particular phenomena in interviews can also be compared with the same phenomena in naturally occurring interactions (Widdicombe, 2025), which is demonstrated in this paper through examining vocabularies of social influence used in both interviews and conference panel discussions.

Over twelve hours of recorded verbal data were collected in total. University research ethics committee approval was given before commencing data collection (University of Edinburgh School of Philosophy, Psychology and Language Sciences Research Ethics Committee approval number 324‐1920). This ensured appropriate measures were taken regarding informed consent, anonymity and data security, following British Psychological Society (2018) ethics guidelines. Sixteen product designers were recruited via a sustainable design LinkedIn group and a design email list to participate in semi‐structured video call interviews conducted by the author between July and October 2020 (see Table 1). The aim was to identify commonalities in ways of accounting for actions across a group of people working in the same profession. The sample includes junior and senior designers and a balance of female and male participants. Participants were told that the interview topic was design decision‐making related to sustainability and that they would be asked to give accounts of recent sustainability‐focused design projects of their choice. The interview guide covered the design process, decisions made, the role of values, and responsibilities. Seven videos of panel discussions at recent sustainable design conferences were selected from YouTube based on the relevance of discussions to designers' roles and decision‐making. This provides naturally occurring conversations in which designers are asked to talk about their roles by peers. Despite the videos being publicly available, details of the specific recordings selected are not provided here to minimize identifiability of the speakers (Joyce et al., 2025).

**TABLE 1:** Participant details.

	Location	Sex	Type of project talked about	Product type	Interview length
1	Germany	M	Professional—in‐house	Trestle table	73:23
2	India	M	Professional—in‐house	Ceiling fan packaging	48:02
3	US/Netherlands	F	Professional—in‐house	Suitcases	45:43
4	Argentina/Italy	M	Professional—independent	Coffee table	65:53
5	UK	F	Internship	Child's bike	41:25
6	UK	F	Student project plus previous work in industry	Cycling backpack & electronics	39:20
7	France	F	Student project plus previous work in industry	Architectural products	42:29
8	Netherlands/ Brazil	F	Student project plus previous work in industry	Plant sensor	29:30
9	US	M	Professional—design agency	Shoe packaging	29:28
10	Spain	M	Design competition	Compost bin	54:13
11	Brazil	M	Professional—independent	Facemask	39:07
12	UK	M	Professional—in‐house	Electric vehicle charge point	37:00
13	US	M	Professional—in‐house	Vehicles	51:40
14	Canada	F	Professional—in‐house	Yoga mat	40:08
15	Germany	F	Professional—independent	Lamp	35:10
16	UK	M	Professional—independent	Plastic cup	59:03

### Analytic procedure

Orthographic transcripts of the full dataset were produced. Across both interviews and panel discussions, a recurring phenomenon of talking about ‘pushing’ was noticed. ‘Pushing’ or ‘push’ appeared 65 times across ten interviews and 17 times across four out of the seven sustainable design conference videos. In most cases, ‘pushing’ was being used to portray efforts to influence others towards more sustainable design decisions. Accounts of educating and teaching others about sustainability were also noticed, representing a different way of influencing. This led to a decision to identify and analyse extracts in which different forms of social influence were constructed. Following standard DP procedure (Wiggins, 2017), 27 extracts in which the designers portrayed their roles as involving influencing were transcribed using Jefferson (2004) notations (see Supporting Information Appendix 1), capturing details such as pauses, changes in pitch and speed, and laughter. These extracts ranged from 12 to 107 lines. Anonymised transcriptions are available via the UK Data Service (Cooper, 2021).

Detailed analysis involved several iterations of closely reading the Jefferson transcripts, making notes on actions, linguistic devices and sequences (Wiggins, 2017). Overall, the accounts of influencing were deemed to serve the purpose of managing expectations that the designers should be doing something about sustainability, despite not being able to make final design decisions. Since sustainability can be considered a collective, moral challenge (Blok et al., 2016; Ha‐Brookshire, 2017), these accounts offer insight into how the designers navigate both moral obligations to influence and also the delicateness of whether they are entitled to seek to change others' actions. Data extracts were discussed at two university data sessions, an established practice in DP research (Wiggins, 2017), and at two conferences, from which peer feedback reinforced the findings as justifiable.

## ANALYSIS

Two contrasting ways of portraying social influence for sustainability were found: constructions of morally normative ‘pushing’ against resistance (31 instances) and constructions of informing and educating to encourage change (28 instances). Specific terms used are listed in Table 2. The analysis explores how the participants evoke these different types of social influence in their depictions, without assuming that they actually used them, and how this represents identity portrayal. Reported speech was found to be used as a way of evidencing both ways of influencing (16 instances). This was most often in the form of self‐reported speech only, with four instances also including reported dialogue between the designer and a manager. Here, five data extracts are shared, selected for the clarity with which they illustrate a particular way of portraying influencing.

**TABLE 2:** Vocabularies used to construct accounts of morally normative and informational social influence.

Type of social influence appealed to	Terms used	Number of instances
Morally normative social influence	Push Keep going Fight Confront Argue Disrupt	31
Informational social influence	Educate Teach Bring ideas/something/a solution Provide options/inputs Give people knowledge Create educational resources Show little examples Get people familiar/comfortable/to hear Inspire	28

Extracts 1 and 2 show designers working up depictions of effortful morally normative social influence. Both are included to demonstrate the similarity of sequencing. Extract 1 is taken from the beginning of an interview with a product designer (P3) who talks about a sustainable design project she was involved in when working for a luggage brand.

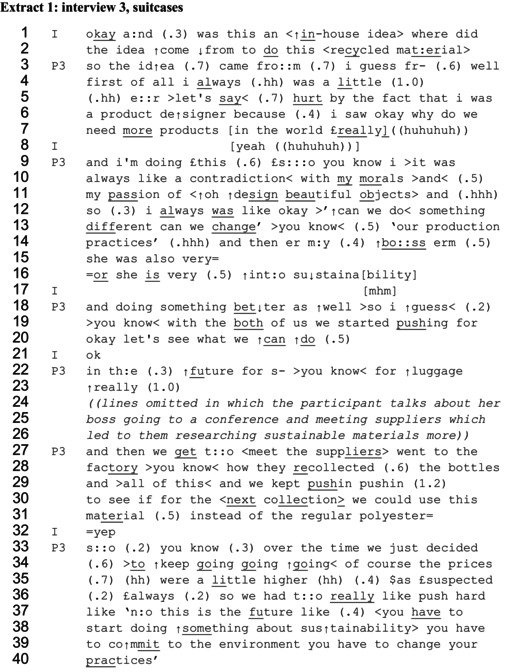

When the interviewer (I) asks where the idea came from to design a more sustainable suitcase (lines 1–2), the designer initially starts to answer, repeating the question wording, saying ‘so the idea came from I guess fr…’ (line 3). Yet there are three fairly long pauses within this utterance, suggesting some difficulty, and indeed she fails to complete this response. Conversation analysis research has shown that a non‐answer is often delivered more slowly (Stivers, 2022). Instead of answering, the designer shifts to portraying identity, constructing sustainability‐related identity in three ways. First, she portrays herself as sensitive to the environmental impacts of design as a whole saying, ‘I always was a little er let's say hurt by the fact that I was a product designer’ (lines 4–6). The use of the extreme case formulation ‘always’ evokes maximal properties to depict this concern as longstanding (Pomerantz, 1986). Second, she makes a contrast, saying ‘it was always like a contradiction with my morals and my passion’ (lines 9–11), portraying a longstanding tension between caring about sustainability as a moral issue and being passionate about design. Third, she uses self‐reported speech or thought to depict questioning whether something can be done to overcome this tension, saying ‘can we do something different can we change you know our production practices’ (lines 12–14). Using the pronoun ‘we’ depicts a collective need to change, includes the speakers' own interest in the outcomes (Edwards & Potter, 1992), and also allows the introduction of her boss in the following lines as also taking part in the influencing.

Once this sustainability‐focused identity has been claimed, the designer describes what she and her manager did to try to make the luggage more sustainable. On lines 19–20 she says, ‘the both of us we started pushing for okay let's see what we can do’, using a metaphor based on physical force (‘pushing’) to represent a psychological process (Gibson, 2019a). What the pushing involves is not specified, nor is what or who is being pushed against. Instead the account focuses on depicting persistent effort and force, using lists and repetition. She says, ‘and we kept pushin pushin to see if for the next collection we could use this material instead of the regular polyester’ (lines 29–31). She continues ‘we just decided to keep going going going’ (lines 33–34), here using a 3‐part repeated list for completeness (Jefferson, 1990). The use of ‘kept’ and ‘keep’, and the repetition of the verbs ‘pushin’ and ‘goin’, depict perseverance, portraying this effort as sustained over time. She strengthens the metaphor by reiterates and upgrading the need for force, saying, ‘so we had to really like push hard’ (line 36). All of these features work to depict the designer and her manager as making significant proactive effort to change design practices, which contributes to the depiction of being a person who cares about sustainability. A possible reason for resistance to the pushing is offered in lines 34–36, through the insertion ‘of course the prices were a little higher as suspected always’. This bracketed comment portrays acknowledgement of a possible economic barrier to focusing on sustainability, but this barrier is minimized by referring to the price increases as only ‘a little higher’.

In the last part of the extract, the designer provides a demonstration of how the pushing is done. She uses self‐reported speech in the form of a three‐part list, a common rhetorical tool often found in persuasion attempts (Jefferson, 1990; Locke & Budds, 2020), saying, ‘[1] you have to start doing something about sustainability [2] you have to commit to the environment [3] you have to change your practices’ (lines 37–40). The three parts of the list all start with the modal verb expression ‘you have to’, depicting the shift to more sustainable practices as an imperative. The use of a non‐specific ‘you’ makes the claim general and works to establish a normative moral order by constructing a shared stake (Edwards & Potter, 1992), as well as avoiding accusing any specific colleagues of not focusing on sustainability. Using ‘you’ rather than ‘we’ here also works to distance the designer from the company when she is highlighting its shortcomings. While reporting the speech of others is typically found to work to add authenticity and credibility to an account (Holt & Clift, 2006), self‐reported speech such as this orients to accountability, evidencing one's own effort to say something (Clift, 2006). Overall, we can infer from the response that sustainability is treated as a moral imperative which entitles the designer and her manager to assertively seek to change others' behaviours.

Extract 2, from an interview with a product designer (P14) who talks about working for a large fitness company, shows a very similar sequence. The participant has been talking about designing a more sustainable yoga mat.

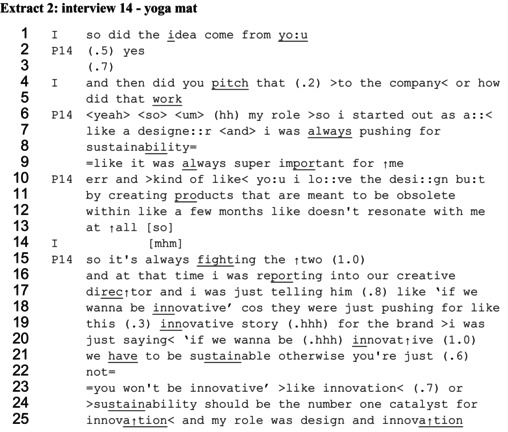

A similar question is asked by the interviewer (I) as in extract 1, ‘so did the idea come from you’ (line 1). There is a preference design in this question, in that it is framed in a way that points to an affirmative answer (Potter, 1996; Schegloff, 2007). This question therefore holds the participant to account for the idea more directly than in extract 1. The response is minimal, ‘yes’ (line 2). There is then a 0.7 s pause before the interviewer seeks an expansion by following up with ‘and then did you pitch that to the company or how did that work’ (lines 3–4). Through this question the interviewer subtly introduces the possibility of the designer engaging in influencing, through ‘pitching’ an idea. The sequence in her response is remarkably similar to that seen in extract 1. After some hesitation (line 5), rather than providing a direct answer, the participant produces general claims about her actions and feelings as a designer, thereby portraying her identity. She says, ‘so I started out as a like a designer and I was always pushing for sustainability’ (lines 6–8). Like in extract 1, the term ‘pushing’ depicts effort, which is different from the interviewer's suggested framing of ‘pitching’. The use of ‘always pushing’ suggests regular action. This regular action is justified in saying ‘it was always super important for me’ (line 9), claiming longstanding personal commitment. An identity tension is portrayed between being a designer and caring about sustainability. She says, ‘I love the design but by creating products that are meant to be obsolete within like a few months doesn't resonate with me at all’ (lines 10–13). The tension is made more explicit in a follow‐up remark in line 15 ‘so it's always fighting the two’.

Her response concludes, again like extract 1, with a section of self‐reported speech, used as a way of evidencing how she sought to influence others. She introduces her self‐reported speech by saying ‘and at that time I was reporting to our creative director and I was just telling him’ (lines 16–17). This portrays her as directly giving advice to her line manager, characterized as a mundane or common occurrence through the use of ‘just’ (also featuring in lines 19–20, ‘I was just saying’) (Lee, 1987). She reports saying to the creative director ‘if we wanna be innovative (…) we have to be sustainable otherwise you're just not, you won't be innovative’ (lines 20–23). The modal verb construction ‘have to’ again marks sustainability as an imperative goal, and the designer depicts appealing to her audience's interest by associating innovation with this goal. She first uses the pronoun ‘we’ in talking about wanting to be innovative and needing to be sustainable as a company, indicating organizational alignment (Whittle et al., 2011) and a collective responsibility. But she then switches to ‘you’ when talking of the consequences of not being sustainable (‘you won't be innovative’, line 23). This pronoun shift works to distance the designer from being accountable for the possible lack of action. This part of the self‐reported speech could be inferred as being a delayed response to the initial question about how she pitched the idea for more sustainable materials. However, she has portrayed forceful pushing, rather than pitching.

In extracts 3 and 4, participants provide accounts of informing and educating, showing orientation to the delicateness of trying to influence others to prioritize sustainability. In extract 3, the participant (P6) continues an extended response to being asked whether designers have a responsibility to do more sustainable design. Prior to the talk in the extract, she had overtly stated that designers ‘actually don't have such a big power’ and that a designer is ‘like a small influencer’. Here, she explains why designers don't have a lot of power and then depicts a way of possibly achieving some influence.

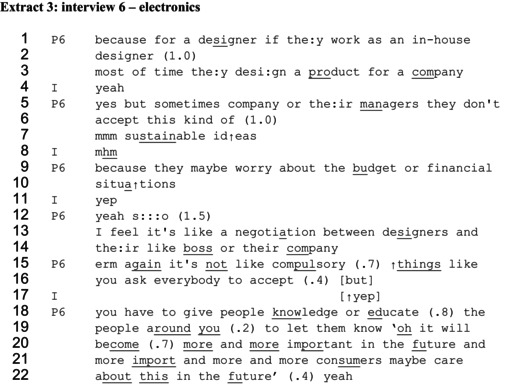

The designer first establishes the context of the claims to follow, in stating, ‘for a designer if they work as an in‐house designer’ (lines 1–2). The challenge of decision‐makers not being persuaded by sustainability arguments is portrayed, saying, ‘but sometimes company or their managers they don't accept this kind of sustainable ideas’ (lines 5–7). The agent enacting the resistance is depicted first as the company and then repaired to the more specific ‘managers’. The designer then cites likely reasons for the managers' resistance, saying ‘because they maybe worry about a budget or financial situations’ (lines 9–10). This portrays her as reasonable and not directly criticizing her managers, through treating the managers as bound by situational factors rather than holding a personal dispositions that are resistant to change (Schegloff, 2005).

The designer then constructs influence as taking place within ‘a negotiation between designers and like their boss or their company’ (lines 13–14). She explicitly states that there is no entitlement to tell others they must prioritize sustainability, saying ‘it's not like compulsory things like you ask everybody to accept’ (lines 15–16). The extreme case formulation (Pomerantz, 1986) ‘everybody’ generalizes those as potentially influenceable, rather than naming specific stakeholders, but also negates any notion that a designer would be entitled to expect their own values to be generalized to all. The actions involved in the aforementioned negotiation are described in two ways, as ‘give people knowledge’ and ‘educate to let them know’ (lines 18–19). In contrast to the depiction of morally justified ‘pushing’ to change someone's actions, these ways of talking about influencing position people as able to receive knowledge, in order to make informed decisions. The extract concludes with the use of self‐reported speech, like in the previous extracts. The self‐reported speech states ‘oh it will become more and more important in the future and more import‐ and more and more consumers maybe care about this in the future’ (lines 19–22). The knowledge to be shared here relates to knowing about consumer preferences. Through claiming a business case for sustainability in future, the designer depicts attending to the economic priorities of the decision‐makers, rather than the moral imperative of sustainability, in an attempt to enable them to decide to prioritize sustainability themselves.

In extract 4, the designer also constructs an account of influencing involving informing decision‐makers, acknowledging economic pressures and managing the delicateness of telling other people what to do. The extract is taken from a response to a question about how the designer felt when working for an architectural company that did not prioritize sustainability.

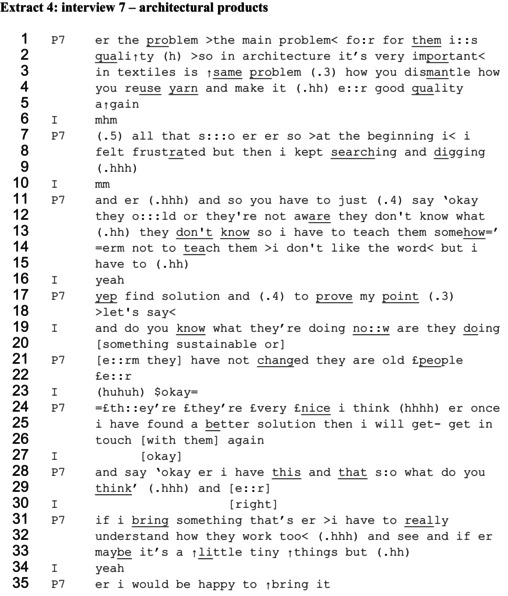

In lines 1–5, the interviewee (P7) establishes that shifting to sustainable design is difficult for the company. She produces a claim ‘the main problem for them is quality’ (lines 1–2), which depicts this statement as a fact, and adds an explanation of the difficulty of reusing materials while maintaining quality. The response portrays the designer as knowledgeable about and understanding of the barriers to making more sustainable choices. She then describes the actions she has taken to find a solution, which works to portray her as committed and persistent. She uses a before and after contrast ‘so at the beginning I I felt frustrated but then I kept searching and digging’ (lines 7–9).

In the rest of the extract, the designer produces a cautious account of educating the company on how to do more sustainable design. Two key features can be identified in how this account is produced. Firstly, she portrays those she is seeking to educate as lacking knowledge. She depicts thinking out loud, using a three‐part list, ‘and so you have to just say ‘[1] ok they are old [2] or they're not aware [3] they don't know what they don't know so I have to teach them somehow” (lines 11–13). The word ‘old’ is extended which emphasises this categorisation, which could be considered to have attributes of not having up‐to‐date professional knowledge. A similar reference to age is made in response to a question about whether the company has shifted towards more sustainable design practices, in saying ‘erm they have not changed they are old people’ (line 21). After laughter from the interviewer, which, rather than signalling something funny, may be working to manage any awkwardness in being dismissive about people due to age (Adelswärd, 1989), the potential offensiveness is to some extent softened by the interviewees' addition of ‘they're they're very nice’ (line 24). Nevertheless, the lack of awareness and knowledge is used as a justification for the designer having to ‘teach’ the people working in the company, which indicates both an ability and entitlement to do so.

However, the second key feature in this account is the hesitation seen in the attempts to describe the actions of educating others. The designer immediately repairs her claim of having to teach them, saying ‘erm not to teach them I don't like the word but I have to yep find solution and prove my point let's say’ (lines 14–18). Claiming to not like the word ‘teach’ is an over‐exposed self‐correction (Bolden et al., 2022), implying there is something problematic about the idea of the knowledgeable designer as teacher and the ignorant company as pupil. The reformulation to finding a solution and proving her point still depicts the passing on of knowledge, but the delicateness of assuming entitlement to do this has been acknowledged.

Like in the previous extracts, the designer then uses self‐reported speech to depict what she might say when attempting to educate, saying ‘ok er I have this and that what do you think’ (lines 28–29). The details of what the solution might be are not provided, instead the focus is on depicting offering possible options and initiating a dialogue. The framing of how knowledge is imparted shifts from teaching and proving her point, to the gentler notion of ‘bringing something’ (lines 31 and 35). She concludes by acknowledging that what she might bring may be only ‘little tiny things’ (line 33) and that her solutions need to fit the context and priorities of the company (‘I have to really understand how they work too’ (lines 31–32)). There is therefore strong orientation to a possible lack of entitlement to assertively influence the company's decisions, and instead a respectful dialogue is proposed.

Extract 5 is taken from a sustainable design online conference panel discussion and shows similar portrayals of ways of influencing as a designer. An electronics designer (D) working within a large company, who has just given a presentation about his work to develop more sustainable products, responds to a question about decision‐making. In his response, he gives an account of social influence including both informing and inspiring, and ‘pushing’. An audience member had typed a question in the chat window, which was paraphrased by the panel chair (C), asking who makes the design decisions.

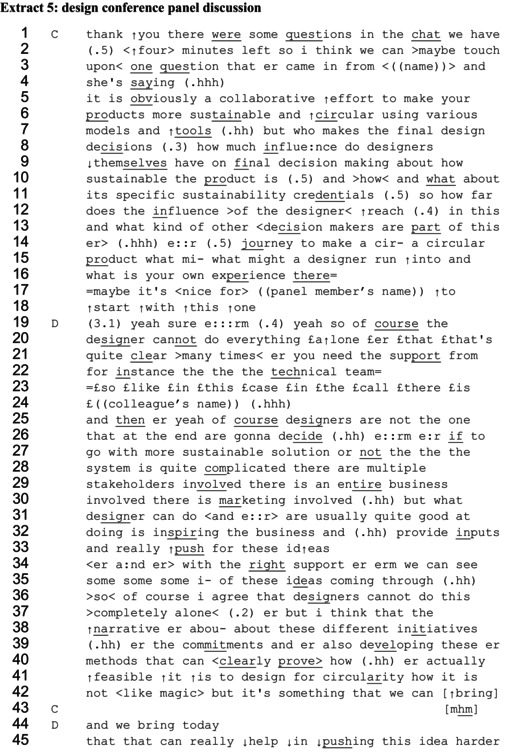

The detailed question which contains several parts, including asking how much influence designers have over decisions related to the sustainability of products, given the multistakeholder character of design. The analytical remarks here focus on how the designer describes influencing in his response. There is first a pause of longer than three seconds, suggesting difficulty (Bilmes, 2014), and also an elongated ‘erm’ (line 19). The designer starts by providing agreement with the description of design as collaborative. He says, ‘yeah so of course the designer cannot do everything alone’ (lines 19–20). The ‘of course’, reflecting the ‘obviously’ in the initial question, depicts this as common knowledge among those present, demonstrating assumed alignment (Kaneyasu, 2021). After reiterating the limited power of designers to decide, his response then focuses on describing what designers *can* do. He lists three behaviours that he claims characterize the capabilities of designers in general, as first ‘inspiring the business’, then to ‘provide inputs’ and then ‘really push for these ideas’ (lines 32–33). The first two items on the list resonate with the interview participants' depictions of educating as a way of influencing that takes into account others' agency to make informed decisions, while the third, ‘push’, reflects other interview participants' accounts of assertive influencing in the face of implied resistance.

In the next part of the extract, the designer again acknowledges the limits to what designers can do but proposes that sustainable design is a feasible goal. He refers to a ‘narrative about’ ‘initiatives’, ‘commitments’ and ‘methods’ (lines 38–40) which he argues can ‘clearly prove’ (line 40) the feasibility. This comment works to demonstrate knowledge of a repertoire of pre‐existing approaches to make design more sustainable and portrays influencing as involving primarily providing solutions and information. Yet at the end of the extract, the designer reiterates the idea of pushing. He claims that the things designers can bring ‘can really help in pushing this idea harder’ (line 45). When speaking about designers' roles in general, rather than in a specific project, the designer is able to construct general educating and pushing as two elements of the same strategy of social influence.

## DISCUSSION

This study contributes to understanding the situated use of the psychological thesaurus to navigate identity and entitlement when portraying social influence. Sustainability‐focused product designers' accounts are analysed to understand how they conceptualize different strategies for influencing for sustainability, and how these relate to psychological theories of social influence. Since these accounts are constructed within the context of interviews or conference panel discussions, flexible identity management is the core action undertaken (Antaki & Widdicombe, 1998). Depicting influencing, in the absence of power to decide, provides a way of claiming to be an active, concerned professional with regard to sustainability. The findings provide insights into the specific interactional phenomena seen in this identity work.

The main finding of this study is that two distinct sets of vocabularies are used to depict social influence as either morally normative, orienting to a clear entitlement to ‘push’ for change, or based on sharing information that may (or may not) influence others' decisions, orienting to the delicateness of trying to influence another's actions. These additional vocabularies of social influence build on Humă et al.'s (2020a) work, through highlighting the frequent use of the verb ‘to push’, as well as phrases such as ‘bringing ideas', ‘proving’ and ‘providing options'. Unlike traditional theories of normative and informational social influence that focus on the influencee's motivations (Deutsch & Gerard, 1955; McDonald & Crandall, 2015), in the designers' accounts, social influence is portrayed from the perspective of the influencer. The different sets of vocabularies demonstrate different ways of negotiating a tension between moral values and professionalism, involving appealing to either moral norms or factual information.

The accounts of ‘pushing’ provide novel insights into the construction of morally normative social influence in the context of identity management. The designers who construct ‘pushing’ for sustainability do not appeal to social norms by depicting what others do, as in the psychological definition of normative (Nolan et al., 2008), but focus on constructing sustainability as an obvious, shared moral obligation. They can thus construct assertive influencing for sustainability, including of more senior stakeholders, without expecting others in the interactions to question their entitlement to do so. The idea that people should behave in certain ways can be a moral imperative, rather than a social convention (Debru, 2011). The designers can be said to be appealing to morally normative social influence, aligning with the definition of normative in philosophy and economics as relating to outcomes that are considered morally desirable, correct or ideal (Debru, 2011; Weston, 1994).

Indeed, the need for a more comprehensive understanding of *normative* in social psychology has previously been raised. For example, Cialdini et al. (1990) note that normative can mean different things and advocate more clarity on norms in experimental studies. They distinguish between injunctive norms (how others think one should behave) and descriptive norms (what others do) (Cialdini et al., 1990). In the data, participants could be said to construct a moral, injunctive norm of sustainability as a norm shared by designers as a group. The norm constructs expectations of how designers should influence decision‐makers and how company decision‐makers should act. Field experiments on normative social influence for sustainability (Nolan et al., 2008; Schultz, 1999) should explicitly acknowledge the morally normative dimension of persuading others to take action for the environment, alongside compliance effects of portraying such action as a social norm.

The accounts of informing and educating about sustainability have parallels with Deutsch and Gerard's (1955, p. 629) theory of informational social influence, defined as ‘an influence to accept information obtained from another as evidence about reality’. In these instances, the participants depict providing information to enable people to make their own informed decisions (thereby constituting persuasion), constructing a more cautious orientation to moral obligation and entitlement. The designers are appealing to the idea of influencees changing their behaviour based on trusted sources of information, while acknowledging competing situational factors. The designers claim a K+ stance, knowing more about the issue, while depicting the company colleagues as knowing less, and so having a K‐ stance, drawing on Heritage and Raymond's (2005) terminologies for epistemics in talk. This is most evident in extract 4 when people are described as being old, unaware and not knowing, and so potentially needing, teaching. This helps maintain the designers' identities as the experts being interviewed about sustainability in design.

In Humă et al.'s (2020a) study, lack of entitlement to influence related to the idea of trying to persuade someone to do something they may not want to do. In the present study, it relates to recognizing possible economic constraints in workplace decision‐making, which are seen to limit how much action can be taken by businesses on sustainability. Echoing Whittle et al.'s (2011) findings on workplace change processes, the speakers navigate both the imperative for change and the wider interests of the people to be influenced, which may justify their resistance. This also resonates with Billig (1988) studies of ideological dilemmas in everyday life, showing how people find subtle ways of telling superiors what to do, using politeness to soften language and avoid appearing coercive. Through depicting cautiousness and consideration of other interests in the accounts of influencing, the participants depict themselves as reasonable people who take a balanced approach rather than an activist stance. While the goal of sharing information may be inferred to be persuasion, the designers depict influencees as being entitled to resist based on external factors, whether they are personally persuaded or not.

Two further findings make contributions to the understanding of discursive strategies for identity management used when talking about social influence. First, the different contexts of the accounts involve differing identity management strategies. Interview participants tend to construct their influencing approaches as either morally normative pushing or tentative informing while maintaining professional alignment. Yet the designer on the conference panel combines both, balancing professionalism and moral commitment when speaking to an audience of peers. Interviewees orient to the interviewer's questions, the interview purpose and assumptions about the interviewer's stance on sustainability (Antaki & Widdicombe, 1998; Rapley, 2015), while panel speakers, addressing a large public audience, need to attend to multiple expectations of being a professional designer, since their public reputation is at stake.

Second, self‐reported speech is frequently found to be used to strengthen depictions of both morally normative influencing and tentative informational influencing, through enacting how the designers may influence others. This aligns with Myers' (1999) findings that people use hypothetical reported speech to enact how they deal with possible opposition. Clift (2006) found that where self‐reported speech occurs in interactions follows specific patterns. In the present study this is indeed the case, as the self‐reported speech is found at the end of a turn, after establishing the personal motivations for influencing and then describing the influencing approach. The self‐reported speech device is used to evidence the designers' effort to say something that could convince others to take action on sustainability. While reporting the speech of others is typically seen as being used to corroborate claims (Holt & Clift, 2006), self‐reported speech helps bring to life an account of what the speaker themselves claims to have said. Self‐reported speech remains underexplored compared with reported speech of others (Clift, 2006). Future studies could further explore its use in accounts of influencing.

As a study of situated interactions, these findings are not meant to be generalisable across all contexts, yet there are implications for understanding and researching social influence in other settings. Prior DP findings have been found to be potentially transferable across similar interactional contexts (Goodman, 2008). The frequency of use of ‘pushing’ for sustainability in both interviews and conference discussions is notable. This metaphor of effort and physical force might appear in different contexts in which people describe their influencing towards a moral goal. It may therefore be appropriate for researchers who are asking participants about influencing for such wider goals in organizations, to use the participants' term ‘pushing’, rather than terms grounded in psychological theories (Edwards, 2012).

The portrayal of the two different ways of influencing for sustainability in organizations found in this study resonates with several other bodies of research beyond psychology. Organization studies research on the strategies reportedly used by insider change agents to sell issues such as sustainability to colleagues and managers has frequently highlighted how sustainability can be framed as either bringing business benefits or as a moral imperative (Alt & Craig, 2016; Dutton & Ashford, 1993). Research on environmental activism has found activists can use either a collaborative or confrontational approach (Büchs et al., 2015; Geels et al., 2015; Kirsop‐Taylor et al., 2023). DP can contribute further analysis of ways of both talking about and accomplishing social influence for sustainability, to better understand how people orient to identity tensions, entitlement and moral and social norms in interactions.

## CONCLUSION

The topic of influencing for sustainability receives a lot of attention in business management and environmental activism literature but requires further exploration in social psychology. This study uses DP to provide novel insights into how social influence for sustainability is portrayed in product designers' accounts of their work, from both research interviews and conference panel discussions. Designers construct accounts of their efforts to influence decisions, thereby managing their identities as people committed to and taking action on environmental protection.

Two distinct ways of depicting influencing are identified: (1) morally normative social influence, associated with a moral obligation and entitlement to assertively influence others to prioritize sustainability, and (2) informing and educating to enable others to make their own decisions, claiming less moral entitlement to influence others' behaviours, and acknowledging competing priorities. Distinct vocabularies of social influence (Humă et al., 2020a) are used to depict these two different ways of influencing. The designers are shown to manage the delicate balance between acting on moral values and maintaining professionalism through the ways they depict influencing. Parallels with theories of normative and informational social influence are identified, contributing an alternative framing of normative social influence appealing to moral norms rather than only social norms. Self‐reported speech is found to be frequently used to evidence taking action to influence others, in accounts of both morally normative and informational social influence. The findings demonstrate different strategies for managing identity tensions, accountability for taking action and entitlement to act, when asked to account for one's actions regarding prioritizing sustainability at work.

## AUTHOR CONTRIBUTIONS

**Liz Cooper:** Conceptualization; investigation; funding acquisition; writing – original draft; methodology; writing – review and editing; formal analysis; project administration; data curation.

## CONFLICTS OF INTEREST

The author declares that there are no Conflicts of Interest related to this research.

## Supporting information


**Appendix 1:** Transcription symbols (Wiggins, 2017, adapted from Jefferson, 2004)

## Data Availability

The data that support the findings of this study are available via the UK Data Service at https://doi.org/10.5255/UKDA‐SN‐855100.
