# Supplementary material for: Identity and entitlement in accounts of (morally) normative and informational social influence for sustainability
Source: Br J Soc Psychol. 2026 Mar 8;65(2):e70061. doi: 10.1111/bjso.70061 (PMC12967718; doi:10.1111/bjso.70061)
Supplement: Supplementary file 1 — Appendix 1: Transcription symbols (Wiggins, 2017, adapted from Jefferson, 2004) [file BJSO-65-0-s001.docx]

**Appendices**

Appendix 1: Transcription symbols (Wiggins, 2017, adapted from Jefferson, 2004)

| (.) | A micro-pause around one tenth of a second |
| --- | --- |
| (1.2) | A pause or silence, measured in seconds and tenths of seconds |
| = | Latched talk, where there is no hearable gap between words (can occur within a turn at talk, or between speakers) |
| :: | Stretched sounds in talk; the more colons, the longer the sound, as in rea::lly l:::ong sounds |
| CAPITALS | Talk that is noticeably louder in contrast to the surrounding talk (sometimes shouting) |
| Underlined | Emphasised words, or parts of words, are underlined |
| ° | Degree symbols enclose noticeably °quieter° talk, with double degree signs indicating °°whispering°° |
| > < | ‘Greater than’ and ‘less than’ symbols enclose talk that is at a faster pace (>speeded-up< talk) than the surrounding talk |
| < > | ‘Less than’ and ‘greater than’ symbols enclose talk that is at a slower pace (<slowed down> talk) |
| ↑ ↓ | Upward arrows indicate a rising pitch in talk, downward arrows indicate falling pitch |
| £ | British pound sign indicates smiley voice or suppressed laughter |
| # | Hashtag indicates ‘creaky’ voice such as when someone is upset. |
| [ ] | Square brackets indicate the start (and end) of overlapping talk |
| Hh | Hhs indicate audible breaths. A dot followed by hs (.h) indicate audible inbreaths; without the dot (as in hh) is an outbreath. Within a word (as in ‘ye(h)s’), this indicates laughter while talking (‘interpolated laughter’). The more hs, the longer the breath. |
| Huh/heh/hah | Laughter can be represented with outbreaths that have vowel sounds within them. |
| *‘yes’* | Single quotation marks are used to indicate reported speech or thought |
| *(( ))* | Double brackets (sometimes without italics) contain details about other features that have not been transcribed, e.g., *((waves hand))* |
| (Unclear) | Words in single brackets are the transcriber’s best guess at what was being said, or (unclear) or (inaudible) if it really can’t be heard clearly |
